# The Flavonoid Luteolin Inhibits Fcγ-Dependent Respiratory Burst in Granulocytes, but Not Skin Blistering in a New Model of Pemphigoid in Adult Mice

**DOI:** 10.1371/journal.pone.0031066

**Published:** 2012-02-06

**Authors:** Eva Oswald, Alina Sesarman, Claus-Werner Franzke, Ute Wölfle, Leena Bruckner-Tuderman, Thilo Jakob, Stefan F. Martin, Cassian Sitaru

**Affiliations:** 1 Allergy Research Group, Department of Dermatology, University Freiburg Medical Center, Freiburg, Germany; 2 Molecular Dermatology, Department of Dermatology, University Freiburg Medical Center, Freiburg, Germany; 3 Competence Centre Skintegral, Department of Dermatology, University Freiburg Medical Center, Freiburg, Germany; 4 Faculty of Biology, University of Freiburg, Freiburg, Germany; 5 BIOSS Centre for Biological Signalling Studies, Freiburg, Germany; 6 Freiburg Institute for Advanced Studies, Freiburg, Germany; University of Tübingen, Germany

## Abstract

Bullous pemphigoid is an autoimmune blistering skin disease associated with autoantibodies against the dermal-epidermal junction. Passive transfer of antibodies against BP180/collagen (C) XVII, a major hemidesmosomal pemphigoid antigen, into neonatal mice results in dermal-epidermal separation upon applying gentle pressure to their skin, but not in spontaneous skin blistering. In addition, this neonatal mouse model precludes treatment and observation of diseased animals beyond 2–3 days. Therefore, in the present study we have developed a new disease model in mice reproducing the spontaneous blistering and the chronic course characteristic of the human condition. Adult mice were pre-immunized with rabbit IgG followed by injection of BP180/CXVII rabbit IgG. Mice pre-immunized against rabbit IgG and injected 6 times every second day with the BP180/CXVII-specific antibodies (n = 35) developed spontaneous sustained blistering of the skin, while mice pre-immunized and then treated with normal rabbit IgG (n = 5) did not. Blistering was associated with IgG and complement C3 deposits at the epidermal basement membrane and recruitment of inflammatory cells, and was partly dependent on Ly-6G-positive cells. We further used this new experimental model to investigate the therapeutic potential of luteolin, a plant flavonoid with potent anti-inflammatory and anti-oxidative properties and good safety profile, in experimental BP. Luteolin inhibited the Fcγ-dependent respiratory burst in immune complex-stimulated granulocytes and the autoantibody-induced dermal-epidermal separation in skin cryosections, but was not effective in suppressing the skin blistering *in vivo*. These studies establish a robust animal model that will be a useful tool for dissecting the mechanisms of blister formation and will facilitate the development of more effective therapeutic strategies for managing pemphigoid diseases.

## Introduction

Pemphigoids are autoimmune blistering disorders associated with autoimmunity against hemidesmosomal proteins [1]. Collagen XVII (CXVII) is a major autoantigen in different pemphigoid diseases, including bullous pemphigoid (BP), pemphigoid gestationis, linear IgA disease, mucous membrane pemphigoid and lichen planus pemphigoides [1]. BP is a prototypical organ-specific autoimmune diseases of the skin associated with subepidermal blisters and autoimmunity against the hemidesmosomal proteins CXVII and BP230 at the dermal-epidermal junction (DEJ) [1], [2]. While the plakin protein BP230 is an intracellular hemidesmosomal component [3], [4], CXVII also known as the bullous pemphigoid antigen of 180 kDa (BP180) is a transmembrane collagen with its N-terminus intracellularly located, its ectodomain spanning the lamina lucida, and its C-terminus reaching the lamina densa of the basement membrane [5], [6]. The ectodomain of BP180/CXVII consists of 15 interrupted collagenous regions and contains major epitopes of pemphigoid autoantibodies and autoreactive T cells within its 16^th^ non-collagenous (NC16A) region [7]–[9]. Further antigenic determinants in pemphigoid diseases were shown on both intra- and extracellular domains of BP180/CXVII [10], [11].

The pathogenic significance of autoantibodies against BP180/CXVII is supported by several lines of evidence: (1) serum levels of circulating autoantibodies against BP180/CXVII correlate with disease activity in patients with BP [12]–[15]; (2) pemphigoid autoantibodies and rabbit antibodies generated against BP180/CXVII recruit leukocytes to the DEJ and induce dermal-epidermal separation of human skin [16], [17]; (3) rabbit antibodies generated against BP180/CXVII induce subepidermal blistering when injected into mice and hamsters [18], [19]; (4) the passive transfer of autoantibodies from BP patients into CXVII-humanized mice induces dermal-epidermal separation [20], [21]; (5) the transfer of maternal antibodies against human BP180/CXVII induces spontaneous skin blistering in pups humanized for the autoantigen [22]; (6) the transfer of splenocytes from mice immunized against human BP180/CXVII into Rag2^−/−^/CXVII-humanized mice results in a sustained production of blister-inducing autoantibodies, partially mimicking the features of the disease in humans [23]. While the pathogenic role of autoantibodies to BP230 was suggested by several observations in patients and experimental animals, the contribution of anti-BP230 reactivity to disease pathogenesis still needs further investigation [15], [24]–[26].

Previous attempts to induce blistering by injecting BP patient IgG into wildtype mice have failed [27], [28]. To circumvent this problem, elegant alternative models have been developed using the passive transfer of antibodies generated against the murine autoantigens or, more recently, by the use of patients' autoantibodies in transgenic mice expressing human BP180/CXVII [18]–[21]. These ingenious models reproduce most of the major disease features and offer unique opportunities to elucidate the pathomechanisms underlying autoantibody-induced tissue damage in pemphigoid diseases. However, major shortcomings of these models are: 1) the fact that neonatal mice injected with BP180/CXVII-specific antibodies do not develop spontaneous skin blistering and 2) by their experimental design, the models in neonatal mice have a short observation period and do not allow for adequately reproducing the course of the chronic pemphigoid disease for pathogenic and therapeutic studies.

Therefore, in the present study we set out to develop a pemphigoid disease model in adult mice reproducing the spontaneous blistering and the chronic course characteristic of human condition. In a model of progressive antibody-induced glomerulonephritis it has been shown that pre-immunization with rabbit IgG in complete Freund's adjuvant several days prior to injection of rabbit anti-mouse glomerular basement membrane (GBM) antiserum results in strong inflammation with deposition of IgG and complement C3 and glomerular lesions [29]. Using this information, we pre-immunized mice with rabbit IgG before passively transferring rabbit IgG generated against BP180/CXVII. Mice pre-immunized against rabbit IgG and repeatedly injected with the BP180/CXVII-specific IgG developed spontaneous sustained blistering of the skin. In addition, blistering was associated with complement activation and recruitment of inflammatory cells and partly dependent on Ly-6G-positive cells. In further *ex vivo* and animal experiments, we investigated the therapeutic potential of luteolin, a plant flavonoid with potent anti-inflammatory and anti-oxidative properties [30]. Luteolin inhibited the Fcγ-dependent respiratory burst in immune complex (IC)-stimulated granulocytes and the autoantibody-induced dermal-epidermal separation in skin cryosections, but was not effective in suppressing the skin blistering in the new animal model of BP.

## Results

### Generation and characterization of rabbit antibodies against murine BP180/CXVII

Rabbit antibodies were generated against different fragments of murine BP180/CXVII by immunizing the animals with a mixture of the respective recombinant proteins. These antibodies were shown to bind to the basement membrane zone of murine skin by indirect immunofluorescence (IF) microscopy (**Figure S1A and B**). By immunoblotting, we showed that the generated antibodies recognized a 180 kDa protein band in extracts of BP180/CXVII-expressing COS-7 cells (**Figure S1C**).

### Complement-activating capacity of rabbit IgG specific for murine BP180/CXVII

**Figure 1:**
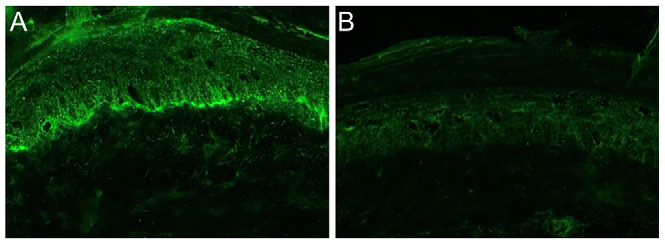
Complement-binding capacity of BP180/CXVII-specific rabbit antibodies. Frozen murine skin sections were incubated with rabbit antibodies and subsequently with fresh human serum as a source of complement. Bound C3 was visualized at the dermal-epidermal junction by fluorochrome-labeled antibody. (**A**) Deposits of C3 in sections incubated with BP180/CXVII-specific rabbit antibody. (**B**) No C3 deposition in sections incubated with normal rabbit IgG (magnification, ×400).

The complement-activating capacity of BP180/CXVII-specific IgG was evaluated by an *in vitro* complement-binding test (**Figure 1**). BP180/CXVII-specific IgG (**Figure 1A**), in contrast to normal rabbit IgG (**Figure 1B**), fixed complement at the epidermal basement membrane.

### Granulocyte-activating capacity of rabbit IgG specific for murine BP180/CXVII

**Figure 2:**
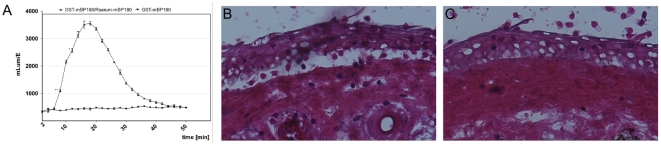
*Ex vivo* granulocyte-activation capacity of BP180/CXVII-specific rabbit IgG antibodies. (**A**) Leukocytes (3×10^7^/ml) were stimulated with rabbit ICs consisting of 5 µg recombinant BP180/CXVII/well and 100 µl of 50-fold diluted BP180/CXVII-specific rabbit serum. ROS production was measured over a period of 60 min. Data are represented as mean ± SD; p<0.001. (**B, C**) Frozen skin sections were incubated with IgG and with 3×10^7^ leukocytes/ml. Dermal-epidermal separation was observed in sections treated with (**B**) BP180/CXVII-specific IgG, but not with (**C**) normal rabbit IgG (magnification, ×400).

To analyze the ability of ICs composed of rabbit IgG and murine BP180/CXVII to activate granulocytes, we performed luminol chemiluminescence assays with granulocytes from healthy donors (**Figure 2A**). Incubation of granulocytes with BP180/CXVII-specific IgG complexed with the antigen, but not with the antigen alone, resulted in the production of reactive oxygen species (ROS) (**Figure 2A**). To assess the capacity of BP180/CXVII-specific rabbit antibodies to induce granulocyte-dependent dermal-epidermal separation in human skin, IgG from pre-immune rabbits and animals immunized with murine BP180/CXVII was incubated with skin cryosections, and subsequently with leukocytes. After the addition of leukocytes, BP180/CXVII-specific IgG induced subepidermal splits in skin cryosections (**Figure 2B**), in contrast normal rabbit IgG failed to induce dermal-epidermal separation in cryosections (**Figure 2C**).

### Pre-immunization of mice against rabbit IgG

To accelerate and increase the inflammatory reaction triggered by binding of IgG autoantibodies at the DEJ of murine skin, we pre-immunized mice with purified rabbit IgG mixed with complete Freund's adjuvant. Subsequently, mice were injected with BP180/CXVII-specific or control rabbit IgG. The immunization induced the production of high levels of mouse IgG against rabbit IgG as shown by ELISA (**Figure S2A**). None of the mice pre-immunized with rabbit IgG showed skin disease clinically and histologically nor deposition of mouse IgG or complement C3 at the DEJ. However, injection of mice with BP180/CXVII-specific IgG, but not with normal rabbit IgG, resulted in deposition of mouse IgG at the murine epidermal basement membrane as detailed below.

### Adult mice injected with BP180/CXVII-specific IgG develop spontaneous skin blistering

**Figure 3:**
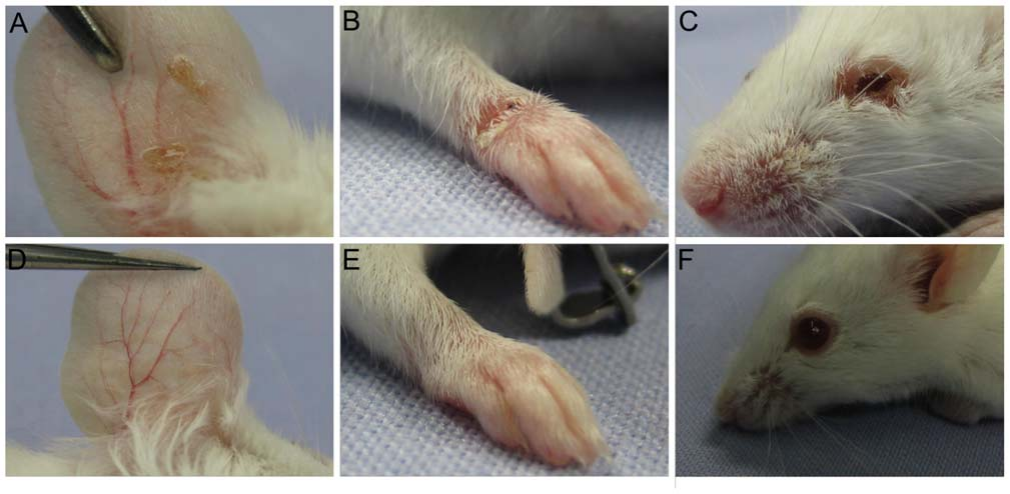
Intraperitoneal injection of BP180/CXVII-specific IgG induces blister formation in adult mice. Skin lesions, including blisters, erosions, and crusts developed on the (**A**) ear, (**B**) front leg and (**C**) periocular area in a BALB/c mouse pre-sensitized with rabbit IgG and subsequently receiving, over a period of 10 days, 6 i.p. injections of IgG, each containing 15 mg of IgG, from a rabbit immunized against murine BP180/CXVII. (**D, E, F**) A control mouse pre-sensitized with rabbit IgG and challenged with the same dose of normal rabbit IgG showed no skin alterations.

**Figure 4:**
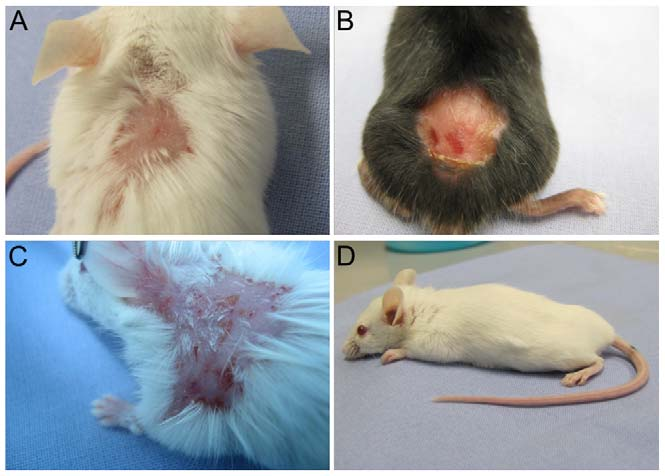
Subcutaneous injection of BP180/CXVII-specific IgG induces blister formation in adult mice. Skin lesions, including blisters, erosions crusts, and alopecia developed in (**A**) BALB/c, (**B**) C57BL6, and (**C**) SJL-1 mice pre-sensitized with rabbit IgG and subsequently receiving, over a period of 10 days, 6 s.c. injections of IgG, each containing 15 mg of IgG, from a rabbit immunized against murine BP180/CXVII. (**D**) A control mouse pre-sensitized with rabbit IgG and challenged with the same dose of normal rabbit IgG showed no skin alterations.

**Figure 5:**
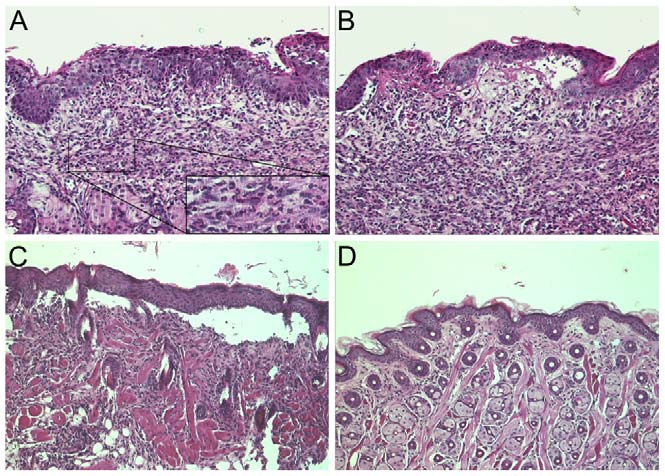
Dermal-epidermal separation in mice injected with BP180/CXVII-specific antibodies. Skin biopsies from mice pre-sensitized with rabbit IgG and subsequently injected with rabbit IgG were stained with hematoxylin and eosin. (**A**) Dense inflammatory infiltrates dominated by granulocytes in a BALB/c mouse receiving BP180/CXVII-specific IgG; (**B**) Subepidermal split and dense inflammatory infiltrates dominated by granulocytes; (**C**) Extensive dermal-epidermal separation; (**D**) Normal histological appearance in a control mouse receiving normal rabbit IgG (magnification, ×400).

Adult SJL (n = 2), BALB/c (n = 31), C57BL/6 (n = 3) and C57BL/10 (n = 2) mice pre-immunized with rabbit IgG were injected intraperitoneally (i.p.) or subcutaneously (s.c.) every second day with 15 mg of IgG purified from rabbit serum for 12 days. Mice (n = 35) injected i.p. (**Figure 3**) or s.c. (**Figure 4**) with IgG from rabbits immunized against murine BP180/CXVII developed skin lesions, including erythema, blisters and erosions. In contrast, mice injected with normal rabbit IgG (n = 5) did not show signs of skin disease (**Figure 3D–F** **and** **4D**). Interestingly, the blistering phenotype induced by the i.p. injection was different when compared with the one triggered by the s.c. injection. While administration of BP180/CXVII-specific IgG by the i.p. route induced blisters at distant sites, including the ears, paws, eyes and snouts (**Figure 3A–C**), the s.c. injections of the BP180/CXVII-specific IgG resulted in lesions mainly restricted to skin areas in the proximity of the injection site (**Figure 4A–C**). Histologically dermal-epidermal separation of the skin with infiltration of inflammatory cells is found (**Figure 5A–C**). Infiltrates were dominated by neutrophils, with few eosinophils (**Figure 5A–B**).

### The blistering phenotype in mice is associated with tissue-bound immunoreactants

**Figure 6:**
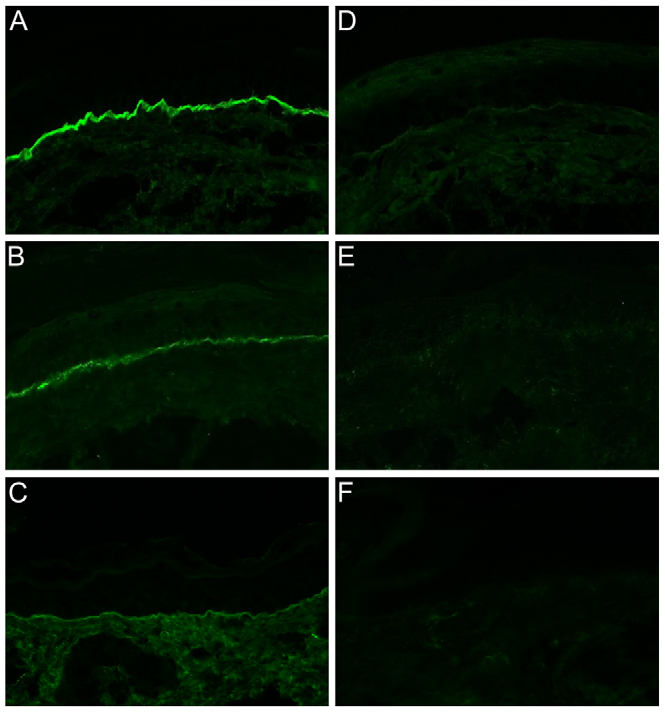
IgG and complement C3 deposition at the basement membrane in experimental bullous pemphigoid. IF microscopy, performed on frozen sections of a perilesional mouse skin biopsy reveals linear deposition of (**A**) rabbit IgG, (**B**) murine C3, and (**C**) murine IgG at the epidermal basement membrane in a diseased mouse. No deposits of (**D**) rabbit IgG, (**E**) murine C3, and (**F**) murine IgG of a control mouse showing no skin lesions (magnification, ×400).

Direct IF microscopy of perilesional skin revealed linear deposits of rabbit IgG (**Figure 6A**) and murine complement C3 (**Figure 6B**) at the epidermal basement membrane in adult mice that received IgG specific for BP180/CXVII. In addition, deposition of murine IgG was found at the basement membrane in mice injected with BP180/CXVII-specific IgG (**Figure 6C**), but not in animals treated with normal rabbit IgG (**Figure 6F**). In mice injected with normal rabbit IgG no deposition of IgG (**Figure 6D**) and complement C3 (**Figure 6E**) was detected by IF microscopy.

### Repeated injection of antibodies induces stable extensive blistering skin disease in mice

**Figure 7:**
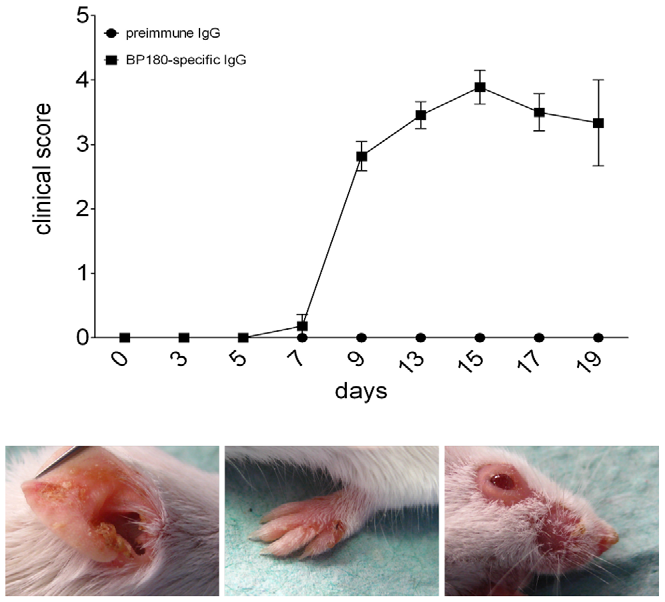
Repeated injections of antibodies against murine BP180/CXVII induce extensive skin blistering. The extent of disease was scored as described in Methods. Means of individual clinical scores of mice injected with BP180/CXVII-specific antibodies (n = 9) and mice injected with normal rabbit IgG (n = 5) are shown before the first injection as well as every subsequent second day for 19 days. The lower panel shows BALB/c mice at day 12 after the first i.p. injection.

In mice injected with rabbit IgG against murine BP180/CXVII, blistering of the skin started at 3–5 days after the first injection and developed during the observation period of 19 days to a full-blown skin blistering disease (**Figure 7**). The extent of the skin disease was scored and serum samples were obtained before the first injection of rabbit IgG (day 0) at day 3 as well as every second day thereafter. Circulating BP180/CXVII-specific rabbit IgG was detected in serum samples by ELISA (**Figure S2B**). During the observation time, disease activity was increasing until it reached a plateau around day 10. Subsequently, while new lesions continued to appear, some areas re-epithelized showing alopecia without scarring.

### Neutrophil depletion partly inhibits blister formation *in vivo*

**Figure 8:**
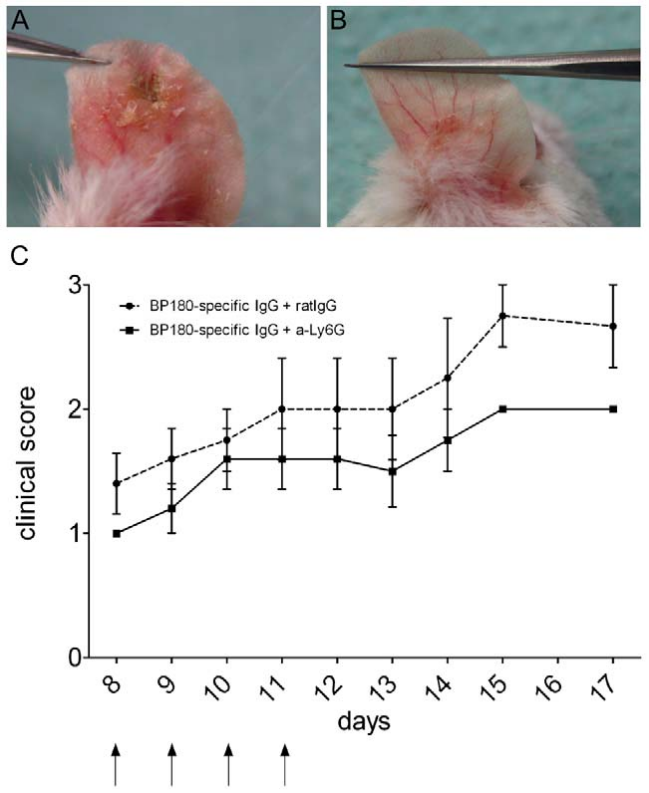
Neutrophil depletion partly inhibits skin blistering induced by BP180/CXVII-specific IgG in mice. Mice pre-immunized with rabbit IgG and injected i.p. with BP180/CXVII-specific rabbit IgG were treated with (**A**) a Ly-6G-specific monoclonal or (**B**) a mock antibody as described in Materials and Methods (arrows). (**C**) Disease activity during the depletion of Ly-6G-positive cells is significantly reduced in the group of mice treated with Ly-6G-specific antibody (n = 5) compared with the group treated with mock antibody (n = 5; p<0.05). Data are shown as mean ± SD.

Since inflammatory infiltrates in our model were mostly composed of neutrophils, we supposed that these cells mediate tissue damage. Therefore, to deplete neutrophils we have used a Ly-6G-specific monoclonal antibody, which induced a severe reduction of neutrophils from around 30% prior to depletion to less than 6% of leukocytes in the peripheral blood. The depletion was maintained for 4 days with a subsequent increase of neutrophils to normal frequency in the peripheral blood within 1 day. Mice pre-immunized with rabbit IgG and injected with rabbit IgG specific for BP180/CXVII developed skin blisters (**Figure 8A and B**). The transient depletion of neutrophils significantly reduced the skin blistering disease in the group of mice treated with the LyG6-specific compared with group treated with the mock antibody (n = 5/group; **Figure 8C**).

### Luteolin inhibits IC-induced ROS production by leukocytes

**Figure 9:**
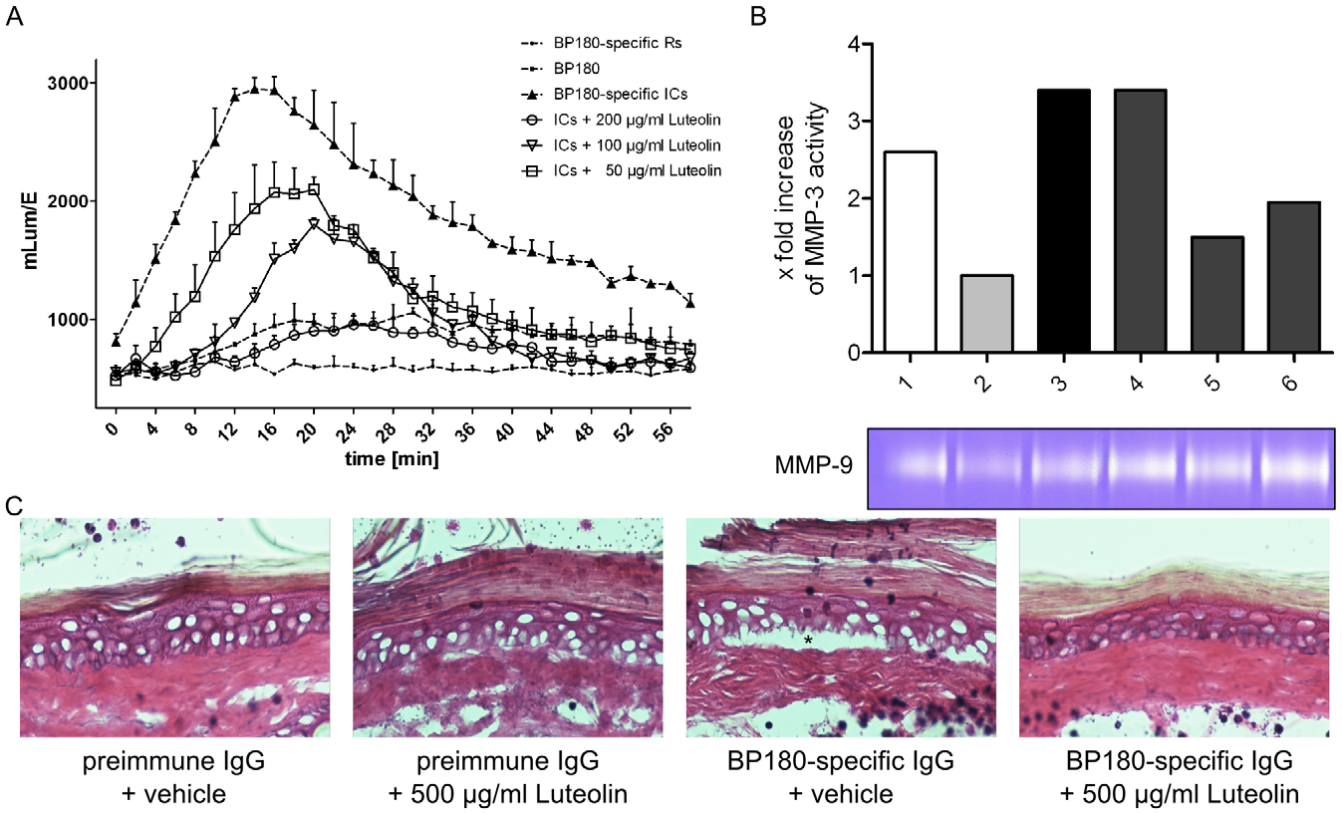
Luteolin inhibits ROS production, but not the release of MMP-9 from IC-stimulated leukocytes. (**A**) Leukocytes (3×10^7^/ml) were pre-incubated with luteolin in different concentrations or vehicle alone and stimulated with rabbit ICs consisting of 5 µg recombinant BP180/CXVII/well and 100 µl of 50-fold diluted rabbit serum. ROS production was measured over a period of 60 min. Data are represented as mean ± SD; p<0.05. (**B**) 3×10^7^/ml leukocytes were stimulated with rabbit ICs consisting of 5 µg recombinant BP180/CXVII/well and 100 µl of 50-fold diluted rabbit serum for 3 h at 37°C. MMP-9 activation was evaluated by zymography in 50-fold diluted supernatants of these cultures of cells stimulated with BP180/CXVII (lane 1), BP180/CXVII-specific rabbit IgG (lane 2), ICs (lane 3), ICs with 1000, 500 and 200 µg/ml Luteolin (lanes 4, 5, and 6 respectively). (**C**) Luteolin inhibits dermal-epidermal separation in cryosection assays *ex vivo*. Frozen murine skin sections were incubated with pre-immune rabbit serum or BP180/CXVII-specific rabbit serum. Significantly less dermal-epidermal separation was observed in sections treated with 500 µg/ml luteolin compared with those treated with vehicle alone (magnification, ×400).

Luteolin, a plant-derived flavonoid with potent anti-oxidative and anti-inflammatory properties, is a promising, small molecule for the treatment in several inflammatory diseases both by systemic and local application [30]. Therefore, we have addressed the effects of luteolin on the autoantibody-induced granulocyte activation and tissue damage in our model. Human leukocytes were stimulated with ICs consisting of BP180/CXVII-specific antibodies and antigen and treated with different concentrations of luteolin (50–200 µg/ml). Under these conditions, we have observed an inhibition of ROS production in a dose-dependent manner (**Figure 9A**). ROS production may play an important role in the secretion of gelatinase B (MMP-9) from activated neutrophils and is believed to be involved in tissue damage in BP. Therefore, we have asked whether luteolin also inhibits the release of MMP-9 from leukocytes stimulated with ICs. We show that the *in vitro* stimulation of human leukocytes for 3 h with ICs and different concentrations of luteolin does not inhibit the production and activation of MMP-9 in the supernatant of these cultures as detected and quantified by gelatine zymography (**Figure 9B**).

### Luteolin inhibits partly autoantibody-induced granulocyte-dependent dermal-epidermal separation *ex vivo*

We have investigated the effects of luteolin on tissue damage using an *ex vivo* cryosection model of autoantibody-induced granulocyte-dependent dermal-epidermal separation. Cryosections were treated either with sera from BP patients and BP180/CXVII-specific rabbit serum or with control sera from healthy donors and pre-immune rabbits followed by incubation with granulocytes from healthy donors. Addition of luteolin, but not vehicle alone, resulted in a significant reduction of the autoantibody-induced dermal-epidermal separation (**Figure 9C**).

### Systemic and local application of luteolin does not significantly impact antibody-induced blistering *in vivo*

**Figure 10:**
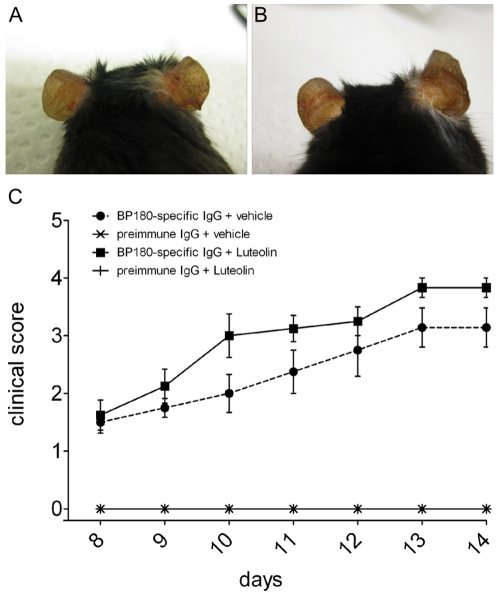
*In vivo* luteolin treatment does not significantly inhibit blistering in adult mice. BALB/c mice were pre-immunized with rabbit IgG and injected s.c. with BP180/CXVII-specific IgG. Control mice were pre-sensitized with rabbit IgG and challenged with the same dose of normal rabbit IgG showed no skin alterations. Topical treatment of mice with (**A**) luteolin at concentrations of 5.24 µM/ear did not result in different outcome compared with (**B**) animals treated topically with vehicle alone. (**C**) Treatment of mice with 1 mg luteolin i.p. daily (n = 8) did not significantly influence disease activity when compared to mice treated with vehicle alone (n = 8, p>0.5). Data are shown as mean ± SEM.

Based on the promising *ex vivo* results, we have extended the investigation of the luteolin effects on the autoimmune injury in our mouse model of BP. We pre-sensitized BALB/c mice with rabbit IgG and injected them every second day s.c. with BP180/CXVII-specific IgG or with pre-immune IgG. These mice were treated every day i.p. either with vehicle alone (n = 8) or with 1 mg of luteolin (n = 8). Luteolin administration did not significantly influence the antibody-induced skin blistering. Mice in both groups showed similar disease scores and antibodies to rabbit IgG throughout the experiment (**Figure 10C** **and Figure S3**). In a subsequent experiment, we have also evaluated the effects of a luteolin-rich topical preparation on the blistering induced in the ears of mice. For this purpose, we have injected s.c. 1.5 mg BP180/CXVII-specific (n = 14) or control IgG (n = 3) into the mouse ears and applied the luteolin-containing RF-40 (n = 10) preparation or vehicle alone (n = 7) every day for 4 days (**Figure 10A and B**). No significant changes were observed in mice topically treated with luteolin compared to those receiving vehicle alone.

## Discussion

Pemphigoid diseases are associated with autoantibodies against BP180/CXVII and BP230. Data from experimental pemphigoid models suggest that BP180/CXVII-specific autoantibodies induce subepidermal skin blistering. Current pemphigoid disease models use neonatal mice injected with antibodies to BP180/CXVII. However, in these models, skin blistering does not occur spontaneously and is only induced when mechanical stress is applied to the skin. In addition, the experimental design using the neonatal mouse model of BP allows for observation periods of only up to 3 days. To address these shortcomings, in the present study we generated a pemphigoid disease model in adult mice pre-immunized against rabbit IgG, which developed a spontaneous extensive blistering skin disease upon injection of murine BP180/CXVII-specific rabbit antibodies and allowed for longer observation times.

The passive transfer of pemphigoid patients' autoantibodies into mice does not result in skin blistering. This is likely due to both a limited cross-reactivity of human autoantibodies with the murine autoantigens and to their lower capacity to activate mouse complement and leukocytes [28], [31]. Therefore, alternative models using the passive transfer of murine BP180/CXVII-specific antibodies generated in rabbits into neonatal mice or neonatal human CXVII-transgenic mice injected with patient IgG have been devised [18], [20], [21]. In a separate study, we could show that purified rabbit IgG antibodies generated against murine BP180/CXVII transferred into adult mice results indeed in spontaneous blistering [32]. However, the disease onset was slower and the overall disease activity lower compared with other models for autoimmune subepidermal blistering disease [33] and typical patients with full-blown disease. To further improve the model, in the present study, we have applied an approach initially devised for the autoantibody-induced glomerulonephritis [29], [34]. This progressive nephrotoxic serum nephritis in mice is a prototypic type II hypersensitivity response in the kidney induced by antibodies directed against the GBM. Pre-sensitization of mice with rabbit IgG prior to challenge with rabbit nephrotoxic serum results in IC formation leading to accelerated glomerular injury and renal dysfunction that resembles aspects of Goodpasture syndrome in humans [29]. In our pemphigoid model in adult mice, pre-sensitization with rabbit IgG resulted in accelerated onset and enhanced activity of the induced pemphigoid disease most likely due to IC formation of mouse anti-rabbit IgG and the tissue-bound rabbit anti-BP180/CXVII.

Our results show the induction of spontaneous skin blisters by the passive transfer of BP180/CXVII-specific IgG. For the induction of subepidermal skin blistering, we used purified rabbit IgG antibodies with high reactivity against the epidermal basement membrane. Similar to autoantibodies from BP patients [35] or to rabbit antibodies against collagen VII [33], our rabbit antibodies fixed complement *ex vivo* as assessed by an *in vitro* complement-binding assay, which measures complement activation by the classical pathway [36]. We also observed complement C3 deposits *in vivo* in our model reproducing the immunopathological finding of C3 deposits in BP patients.

The infiltration of leukocytes, dominated by granulocytes, into the upper dermis of the lesional skin is a further major pathological feature of human BP. We have seen constantly inflammatory infiltrates consisting predominantly of granulocytes in the skin of our mice. We could also show that *in situ*-bound and soluble ICs of rabbit IgG and BP180/CXVII activated granulocytes resulting in respiratory burst and dermal-epidermal separation *ex vivo*. However, while in approximately 50% of BP patients' eosinophils represent the major fraction of recruited cells, in our present model neutrophils were the main cell type observed. Thus similar to previous *in vivo* studies [18]–[21], eosinophil infiltration, an important histopathological feature of human BP, is not fully reproduced in our present model and should be addressed in further studies.

An interesting observation relates to the different clinical phenotypes induced in mice injected with BP180/CXVII-specific IgG depending on the administration route. The i.p. injection of antibodies resulted in generalized skin blistering with predilection on the ears, legs, snout and periocular areas. The s.c. injection of the same IgG preparation triggered extensive skin disease mainly limited to the proximity of the injection area, although a few lesions at distant sites were also observed. The cause of this phenomenon is not known, but is not likely related to a lower systemic availability of pathogenic antibodies, since the levels of circulating BP180/CXVII-specific IgG in the two groups were similar. A further unresolved issue is why lesions develop at predilection sites when the pathogenic IgG is injected intraperitoneally. This site-specificity of the autoimmune attack, which resembles findings in the passive transfer model of epidermolysis bullosa acquisita [33], may be explained by the obvious exposure to increased mechanical trauma and/or by particularities of the vasculature at these sites that were shown to be relevant in antibody-dependent models of rheumatoid arthritis [37].

Results from a neonatal model of BP and our previous data from the *ex vivo* cryosection model of BP showed that granulocyte activation is a prerequisite for autoantibody-induced dermal-epidermal separation [16], [38]. In our present study, depletion of neutrophils using a Ly-6G-specific antibody only partly inhibited skin blistering induced by BP180/CXVII-specific IgG. While a straightforward explanation for the relatively modest effect of neutrophil depletion on skin blistering in the adult model of BP is difficult to give at this time, a combination of factors is likely to have contributed to this outcome. First, the *ex vivo* cryosection model has an obvious limitation due to the fact that the skin sections are dead tissue containing damaged cells precluding more active, keratinocyte-dependent mechanisms to be reproduced in this model. Second, in the neonatal model granulocytes are needed for inducing the “epidermal wrinkling” sign by rubbing the mouse skin. It is however, not known how these cells impact the development of spontaneous blistering reproduced in the new animal model presented here. Importantly, as recently suggested, different mechanisms of autoantibody-induced blister formation may be in place in BP [39], [40], which due to the longer observation time in the new BP model may have become apparent in our present study.

Luteolin (3′,4′,5,7-tetrahydroxyflavone), a plant flavonoid with potent anti-inflammatory and anti-oxidative properties and a good safety profile is being investigated as new therapy in inflammatory autoimmune disease and allergy [30]. Therefore, the aim of a further set of experiments was to investigate the therapeutic potential of luteolin in exprimental BP. Initial *ex vivo* experiments demonstrated that luteolin inhibited the FcγR-dependent production of ROS by leukocytes and partly the pemphigoid autoantibody-induced granulocyte-dependent dermal-epidermal separation of skin sections. However, mice treated with luteolin i.p. or by topical application did not show a lower pemphigoid disease activity upon challenge with blister-inducing rabbit IgG. Taken together these results suggest that luteolin is not acting on key pathogenic mechanisms in experimental BP *in vivo* and therefore do not qualify this compound for further exploring its therapeutic potential for the human pemphigoid disease.

In conclusion, this study demonstrates that the passive transfer of BP180/CXVII-specific IgG antibodies induces spontaneous blistering in adult mice and recapitulates the main clinical, histological, and immunopathological features of human pemphigoid disease. A further salient feature of this model is the fact that it allows longer observation times of the diseased mice. Using this model we could show that granulocytes significantly contribute to the autoantibody-triggered tissue damage. Promising *ex vivo* results using the anti-inflammatory flavonoid luteolin were not validated as effective systemic or topical therapy in the experimental pemphigoid model *in vivo*. These studies establish an animal model that will be a useful tool for dissecting the cellular and molecular mechanisms of blister formation in BP. In addition, this experimental system will facilitate the development of more effective therapeutic strategies for managing this chronic autoimmune disorder.

## Materials and Methods

### Mice

The animal's welfare is checked regularly, and the University Freiburg Medical Center animal facility complies with the national and international laws and regulations. The experiments were approved by the Animal Care and Use Committee (Regierungspräsidium Freiburg, Germany; no. G-10/14 and G-10/84) and performed by certified personnel. 6 to 8-week-old BALB/c, C57BL/6J, C57BL/10 and SJL female mice with a body weight of approximately 20 g were obtained from Charles River, Germany. All injections and bleedings were performed on mice narcotized by inhalation of isoflurane or intraperitoneal administration of a mixture of ketamine (100 µg/g) and xylazine (15 µg/g).

### Generation of antibodies against murine BP180/CXVII

Polyclonal antibodies were produced by immunizing rabbits with 200 µg of a mixture containing purified glutathione S-transferase (GST)-mCXVII-EC1, GST-mCXVII-EC3, GST-mCXVII-EC7 and GST-mCXVII-IC2 in a 2∶1∶1∶1 molar ratio in complete Freund's adjuvant. The recombinant fragments designated GST-mCXVII-EC1, GST-mCXVII-EC3, GST-mCXVII-EC7 and GST-mCXVII-IC2 contain murine collagen XVII sequences stretching from amino acid positions 498–580, 856–901, 1030–1134, and 186–475, respectively [31]. These GST-fusion proteins containing sequences of murine BP180/CXVII were cloned and purified as previously described [31], [41]. Briefly, recombinant GST fusion and His-tagged proteins were expressed in *E. coli* TOP10 and XL1-Blue and purified by gluthatione agarose and metallochelate affinity chromatography, respectively as described [16], [42]. The animals were boosted twice (at 15-day interval) with the same protein preparation in incomplete Freund's adjuvant. Immune sera were obtained at regular intervals and characterized by IF microscopy on cryosections of murine skin.

### Affinity purification of rabbit IgG

IgG from rabbit serum or Amicon Ultra-15 filter-concentrated supernatants from hybridoma cells (clone NIMP-R14) was purified using Protein G Sepharose Fast Flow affinity column chromatography (Amersham Biosciences) as described [43]. Briefly, antibodies were eluted with 0.1 M glycine buffer (pH 2.5), neutralized with 1.5 M Tris-HCl (pH 10), and concentrated under extensive washing with PBS (pH 7.2) using Amicon Ultra-15 filters (Millipore). Purified IgG was filter-sterilized (pore size, 0.22 µm; Millipore) and the protein concentration was measured spectrophotometrically at 280 nm. Reactivity of IgG fractions was analyzed by IF microscopy on murine skin.

### Passive transfer experiments and clinical scoring of disease activity

Adult mice were pre-immunized on day 0 with 200 µg of rabbit IgG and injected starting with day 3 every second day subcutaneously (s.c.) or intraperitoneally (i.p.) six times with 15 mg of rabbit IgG against murine BP180/CXVII with end-titer on adult mouse skin of 1∶16,000–32,000. Control mice received 15 mg of pre-immune IgG purified from rabbits. For neutrophil depletion *in vivo* BALB/c mice were injected i.p. with 0.125 mg of the depleting rat anti-Ly-6G antibody or control rat IgG starting 3 days after injection of BP180/CXVII-specific rabbit IgG on days 5, 7, 8, 9, 10, and 11. Luteolin 98 MM was obtained from International Development and Manufacturing (MMD), dissolved in 1 M NaOH and diluted 1∶10 in PBS to produce a stock solution of 10 mg/ml (pH 8.5). For the luteolin treatment *in vivo*, BALB/c mice were injected i.p. with 1 mg of luteolin dissolved in PBS (pH 8.5) or vehicle alone every day. In mice the LD50 values are of >180 mg/kg by i.p. injection [44]. For topical therapy, mice were injected twice s.c. in the ears with 1.5 mg IgG and the luteolin-rich *Reseda luteola l.* extract (RF-40) was topically used at luteolin concentrations of 5.24 µM/site [45]. All skin samples were examined by IF microscopy for IgG and complement C3 deposition and by light microscopy (H&E staining) for signs of dermal-epidermal separation and/or inflammatory infiltrates, as previously described [46], [47]. Mice were weighed daily and examined for their general condition and for evidence of cutaneous lesions (i.e., erythema, blisters, erosions, and crusts). Intact blisters or erosions were counted and the extent of skin disease was scored as follows: 0, no lesions; 1, fewer than 20 lesions or less than 10% of the skin surface; 2, more than 20 lesions or 10–20% of the skin surface; 3, 20–40% of the skin surface; 4, 40–60% of the skin surface; and 5, more than 60% of the skin surface.

To evaluate the correlation of antibody titers with the extent of disease, sera were obtained from adult mice at different time points, including before starting the treatment and 2 days after the last injection. Sera were assayed for antibody titers by ELISA. Biopsies of lesional and perilesional skin and esophagus were obtained 2–3 days after the last injection and prepared for examination by histopathology and IF microscopy.

### IF microscopy

Indirect and direct IF microscopy using rabbit or mouse sera was performed as described previously [33]. Briefly, indirect IF was carried out on frozen murine skin sections from untreated mice, which were incubated 1 h with 100-fold diluted rabbit serum. Direct IF microscopy was performed using frozen skin sections from treated mice. Bound immunoreactants were visualized using a 100-fold diluted AlexaFluor 488-conjugated antibodies specific for rabbit IgG, mouse C3 and mouse IgG (all Invitrogen). Complement-fixing activity of antibodies to the DEJ was determined as described [43]. Briefly, sections of normal mouse skin were incubated 1 h with 1-fold diluted rabbit sera against BP180/CXVII or control IgG, followed by washing with PBS (pH 7.2), twice for 10 min. Subsequently, sections were treated for 1 h with fresh human serum as a source of complement, diluted 1∶5 with Gelatin Veronal Buffer (Sigma). *In situ* complement deposition was visualized using a 100-fold diluted AlexaFluor-488-conjugated antibody specific for human C3 (Invitrogen).

### ROS measurement by luminol chemiluminescence

Human leukocytes were isolated from the peripheral blood of healthy donors. For the experiments conducted with human leukocytes, we obtained approval from the Ethics Committee of the Medical Faculty of the University of Freiburg, Germany (Institutional Board Project no. 407/08). We obtained written informed consent from the donors whose material was used in the study, in adherence to the Helsinki Principles. After 3% dextran sedimentation erythrocytes were lysed using a hypotonic solution of 0.2% NaCl. Leukocytes were washed in DMEM medium without supplements and resuspended at a final density of 3×10^7^ cells/ml. Cell viability was tested with trypan blue; only preparations with a viability greater than 95% were used. Plate bound murine ICs were formed using the recombinant fragments of murine collagen XVII (GST-mCXVII-EC1, -EC3, -EC7 and -IC2) and rabbit sera from rabbits immunized against BP180/CXVII. Briefly, 96-well microtiter plates were coated with 5 µg of equimolar amounts of GST-mCXVII-fragments in 0.1 M bicarbonate buffer (pH 9.6). The wells were washed five times with PBS (pH 7.2) containing 0.05% Tween 20, after each step. After blocking for 1 h with PBS-Tween containing 1% bovine serum albumin (fraction V; Sigma-Aldrich, Germany), wells were incubated for 2 h with a 50-fold diluted rabbit sera against mouse BP180/CXVII. ROS production was measured using luminol-chemiluminescence. Leukocytes were pre-incubated for 5 min at 37°C with 150 µM luminol (Sigma) and, subsequently, 100 µl of cell suspension was added to each well. To study the effects of luteolin, leukocytes were pre-incubated with luteolin to a final concentration of 50, 100, 200 µg/ml or with vehicle alone for 10 min. All procedures were performed in the dark. Unless otherwise specified the chemiluminescence intensity was recorded continuously for 1 h. Kinetic measurement of ROS production in leukocytes stimulated with PMA (500 nM; Sigma) was conducted as control.

### MMP-9 activity measurement by gelatine zymography

Human leukocytes were isolated from the peripheral blood of healthy donors and treated as described for ROS production. For the analysis of the effects of luteolin on MMP-9 release and activation by IC-stimulated leukocytes, the cells were pre-incubated with luteolin to a final concentration of 1000, 500 and 200 µg/ml or with vehicle alone in DMEM for 3 h at 37°C. The supernatant was collected, diluted 1∶50 in PBS and sample buffer without reducing agents was added. All samples were loaded and separated for 1–2 h on a 8% SDS-PAGE gel containing 1% gelatine under non-reducing conditions. Subsequently, the gel was stained with Coomassie Brillant Blue staining solution for 1 h, followed by destaining the gel for 2 h as described [48].

### Flow Cytometry

Blood samples were lysed with ACK lysis buffer, Fc-blocked with anti-mouse CD16/CD32 and then stained for FACS analysis with anti-mouse Ly-6G-FITC and anti-mouse Gr-1-PE or isotype controls as described previously [49], [50]. Neutrophils were defined as Gr-1^+^ Ly-6G^+^ cells. All antibodies were from BD Pharmingen. FACS analysis was performed with FACS Canto II (Becton Dickinson).

### Cryosection assay

IgG from rabbits or patients was diluted 1∶5 in PBS and the protein concentration was measured using absorbance at 280 nm. Six-micrometer cryosections of murine or human skin were washed in PBS to remove embedding medium and incubated with 100 µl of diluted antibody preparations for 2–3 h at 37°C. Sections were washed twice with PBS and chambers were prepared as previously described [41]. Human leukocytes were isolated from the peripheral blood of healthy donors. For the experiments conducted with human leukocytes, we obtained approval from the Ethics Committee of the Medical Faculty of the University of Freiburg, Germany (Institutional Board Project no. 407/08). We obtained written informed consent from the donors whose material was used in the study, in adherence to the Helsinki Principles. After 3% dextran sedimentation erythrocytes were lysed using a hypotonic solution of 0.2% NaCl. Leukocytes were washed in DMEM medium without supplements and resuspended at a final density of 3×10^7^ cells/ml. Cell viability was tested with trypan blue; only preparations with a viability greater than 95% were used. In some experiments, leukocytes were pre-incubated with luteolin to a final concentration of 1000, 500 or 200 µg/ml or with vehicle alone for 10 min. Leukocyte suspensions were injected into the chambers and incubated for 3 h at 37°C. Chambers were then disassembled and sections washed in PBS, air dried, and stained with hematoxylin and eosin.

### Histology

Biopsies of lesional and perilesional skin and esophagus were fixed in 3.7% buffered formalin. Sections from paraffin-embedded tissues were stained with H&E.

### Immunoblot analysis

For immunoblotting, subconfluent COS-7 cells transformed with murine full length BP180/CXVII cDNA or NIMP-R14 hybridoma cells were lysed for 30 min on ice in a buffer containing 1% Triton X-100, 0.137 M NaCl, 20 mM Tris/HCl pH 8.0 (pH 8.0), 2 mM EDTA, and 1 mM Na_3_VO_4_, 10% glycerol and 1 mM PMSF [33]. The samples were separated by SDS-PAGE on 6% polyacrylamide gels, followed by transfer onto nitrocellulose membrane. 200-fold diluted polyclonal rabbit antibodies directed against murine BP180/CXVII as described above were used. After incubation with a chicken anti-rabbit IgG HRP-conjugated secondary antibody (Novus biologicals), signals were visualized by 3,3′-diaminobenzidine (Merck) [41].

### ELISA

ELISA was used to measure circulating mouse IgG against rabbit IgG following established protocols with minor modification [51]. Briefly, 500 ng purified normal rabbit IgG were immobilized on 96-well microtiter plates using 0.1 M bicarbonate buffer (pH 9.6). After each step, the wells were washed four times with PBS (pH 7.2) containing 0.05% Tween 20, unless otherwise specified. After blocking for 1–2 h with PBS-Tween containing 1% bovine serum albumin (fraction V; Sigma-Aldrich), wells were incubated for 2 h with a 250-fold dilution of mouse sera. Bound antibodies were detected using a 3,000-fold dilution of an HRP-labeled goat anti-mouse IgG antibodies (BioRad) and orthophenylene diamine (Dako) as a chromophore (490 nm).

ELISA was used to measure circulating rabbit IgG against BP180/CXVII following established protocols with minor modification [51]. Briefly, 300 ng of the purified His-tagged murine BP180/CXVII (EC1 fragment) was immobilized on 96-well microtiter plates using 0.1 M bicarbonate buffer (pH 9.6). After each step, the wells were washed four times with PBS (pH 7.2) containing 0.05% Tween 20, unless otherwise specified. After blocking for 2 h with PBS-Tween containing 1% bovine serum albumin (Sigma-Aldrich), wells were incubated for 2 h with a 100-fold dilution of mouse sera. Bound antibodies were detected using a 3,000-fold dilution of a chicken anti-rabbit IgG HRP (NOVUS biologicals) and orthophenylene diamine (Dako) as a chromophore (492 nm).

### Statistical analysis

All analyzes were performed using GraphPad Prism Version 5.03. Continuous variables were compared using the Man-Whitney U or Students' t test. P<0.05 was considered statistically significant.

## Supporting Information

Figure S1 **Reactivity and specificity of IgG antibodies from rabbits immunized with murine BP180/CXVII.** Frozen skin sections were incubated with 100-fold diluted serum from a rabbit (**A**) immunized against BP180/CXVII or (**B**) pre-immune rabbit serum. (**C**) Extracts of the NIMP-R14 hybridoma cell line (*lanes 1 and 3*) and of BP180/CXVII-expressing COS-7 cells (*lanes 2 and 4*) were separated by 6% SDS-PAGE and immunoblottted with 200-fold diluted pre-immune rabbit serum (*lanes 1 and 2*) or serum from a rabbit immunized against BP180/CXVII (*lanes 3 and 4*).
(TIF)

Figure S2 (A) Serum levels of mouse IgG antibodies against rabbit IgG. Levels of murine IgG in serum samples of pre-immunized mice, which were subsequently injected with BP180/CXVII-specific (n = 5) or control (n = 3) rabbit IgG were measured by an ELISA using rabbit IgG as antigen as described in Materials and Methods. Data are shown as mean ± SD. (**B**) **Serum levels of rabbit IgG against BP180/CXVII in mice.** Levels of rabbit IgG autoantibodies in serum samples of mice injected with BP180/CXVII-specific (n = 9) or control (n = 5) rabbit IgG were measured by an ELISA using recombinant BP180/CXVII, as described in Materials and Methods. Data are shown as mean ± SD.
(TIF)

Figure S3 **Luteolin therapy does not influence levels of the injected pathogenic BP180/CXVII-specific IgG and of rabbit IgG-specific mouse IgG antibodies. (A) Serum levels of mouse IgG antibodies against rabbit IgG.** Levels of murine IgG in serum samples of pre-immunized mice, which were subsequently injected with BP180/CXVII-specific (n = 16) or control (n = 11) rabbit IgG and treated with luteolin (n = 14) or vehicle (n = 13) were measured by an ELISA using rabbit IgG as antigen as described in Materials and Methods. Data are shown as mean ± SD. (**B**) **Serum levels of rabbit IgG against BP180/CXVII in mice**. Levels of rabbit IgG autoantibodies in serum samples of mice injected with BP180/CXVII-specific (n = 16) or control (n = 11) rabbit IgG and subsequently treated with luteolin (n = 14) or vehicle (n = 13) were measured by an ELISA using recombinant BP180/CXVII, as described in Materials and Methods. Data are shown as mean ± SD.
(TIF)
