# Supplementary figures and images for: The Flavonoid Luteolin Inhibits Fcγ-Dependent Respiratory Burst in Granulocytes, but Not Skin Blistering in a New Model of Pemphigoid in Adult Mice
Source: PLoS One. 2012 Feb 6;7(2):e31066. doi: 10.1371/journal.pone.0031066 (PMC3273480; doi:10.1371/journal.pone.0031066)

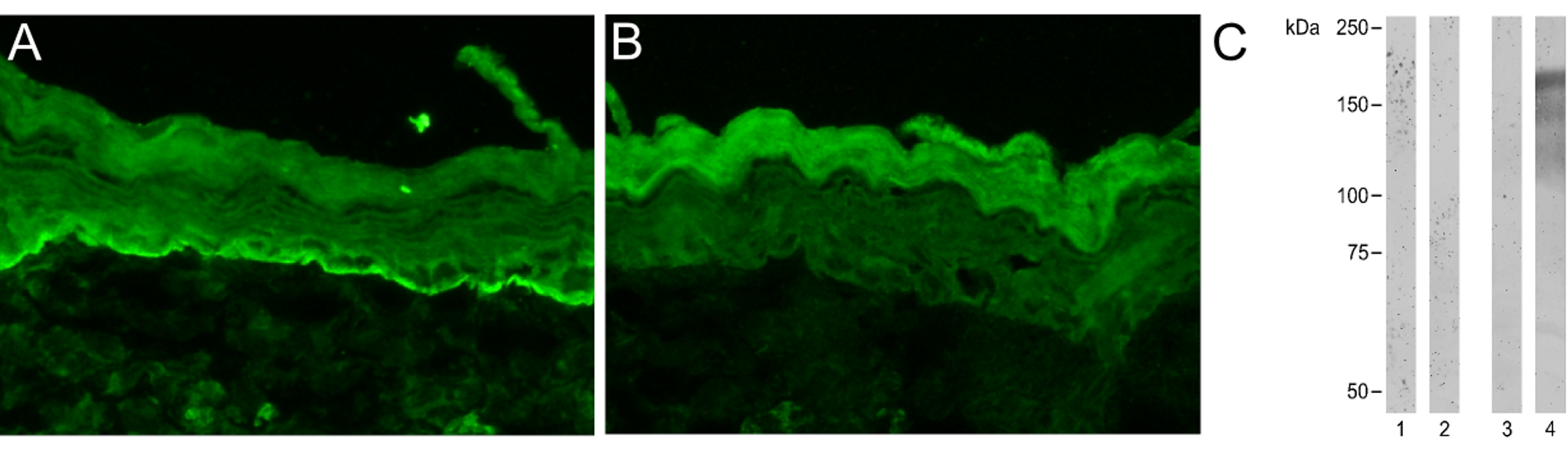

Supplement: Figure S1 — Reactivity and specificity of IgG antibodies from rabbits immunized with murine BP180/CXVII. Frozen skin sections were incubated with 100-fold diluted serum from a rabbit (A) immunized against BP180/CXVII or (B) pre-immune rabbit serum. (C) Extracts of the NIMP-R14 hybridoma cell line (lanes 1 and 3) and of BP180/CXVII-expressing COS-7 cells (lanes 2 and 4) were separated by 6% SDS-PAGE and immunoblottted with 200-fold diluted pre-immune rabbit serum (lanes 1 and 2) or serum from a rabbit immunized against BP180/CXVII (lanes 3 and 4). (TIF) [file pone.0031066.s001.tif]

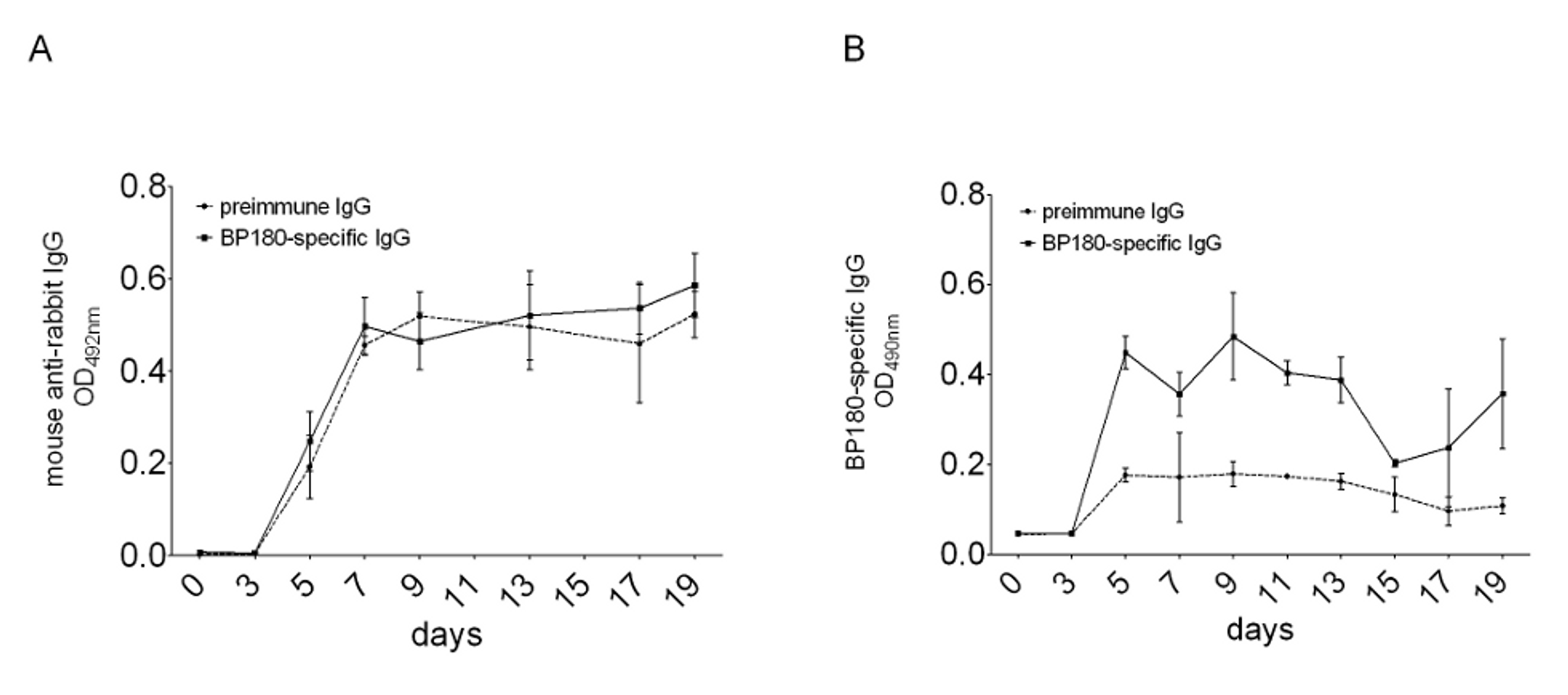

Supplement: Figure S2 — (A) Serum levels of mouse IgG antibodies against rabbit IgG. Levels of murine IgG in serum samples of pre-immunized mice, which were subsequently injected with BP180/CXVII-specific (n = 5) or control (n = 3) rabbit IgG were measured by an ELISA using rabbit IgG as antigen as described in Materials and Methods. Data are shown as mean ± SD. (B) Serum levels of rabbit IgG against BP180/CXVII in mice. Levels of rabbit IgG autoantibodies in serum samples of mice injected with BP180/CXVII-specific (n = 9) or control (n = 5) rabbit IgG were measured by an ELISA using recombinant BP180/CXVII, as described in Materials and Methods. Data are shown as mean ± SD. (TIF) [file pone.0031066.s002.tif]

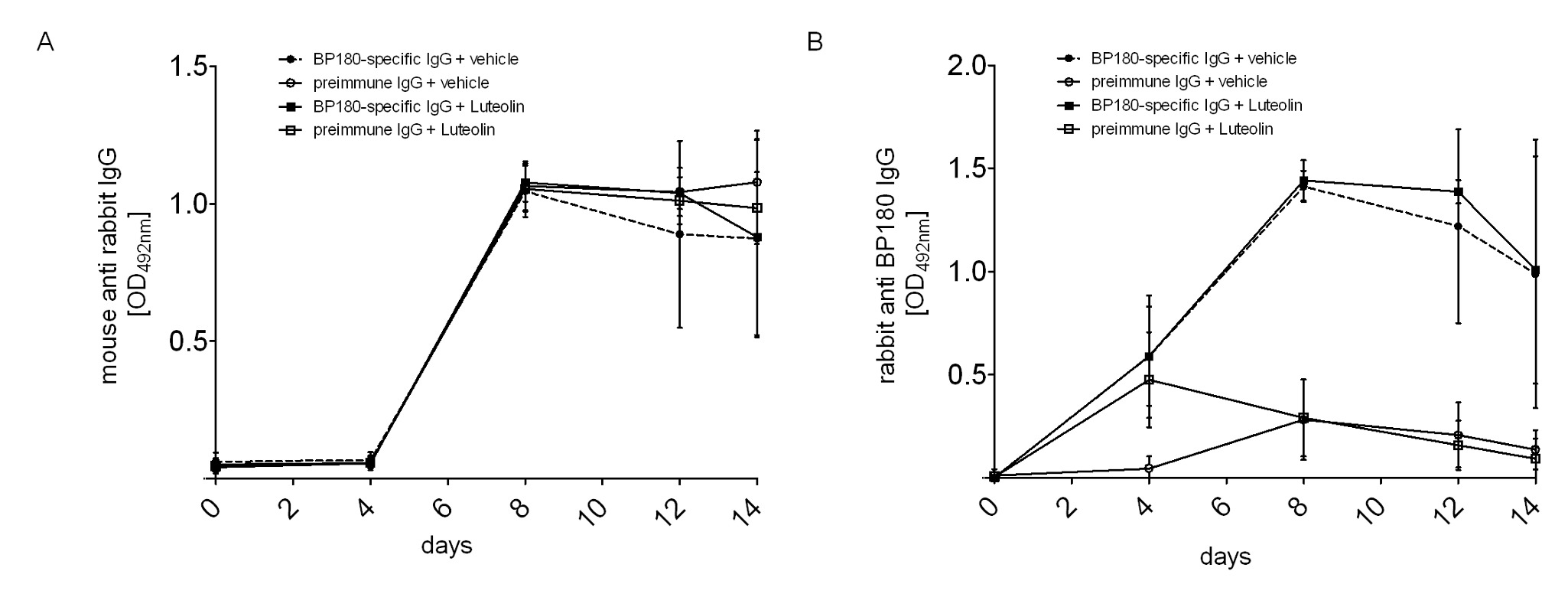

Supplement: Figure S3 — Luteolin therapy does not influence levels of the injected pathogenic BP180/CXVII-specific IgG and of rabbit IgG-specific mouse IgG antibodies. (A) Serum levels of mouse IgG antibodies against rabbit IgG. Levels of murine IgG in serum samples of pre-immunized mice, which were subsequently injected with BP180/CXVII-specific (n = 16) or control (n = 11) rabbit IgG and treated with luteolin (n = 14) or vehicle (n = 13) were measured by an ELISA using rabbit IgG as antigen as described in Materials and Methods. Data are shown as mean ± SD. (B) Serum levels of rabbit IgG against BP180/CXVII in mice. Levels of rabbit IgG autoantibodies in serum samples of mice injected with BP180/CXVII-specific (n = 16) or control (n = 11) rabbit IgG and subsequently treated with luteolin (n = 14) or vehicle (n = 13) were measured by an ELISA using recombinant BP180/CXVII, as described in Materials and Methods. Data are shown as mean ± SD. (TIF) [file pone.0031066.s003.tif]
